# Prevalence and determinants of adherence to HAART amongst PLHIV in a tertiary health facility in south-south Nigeria

**DOI:** 10.1186/1471-2334-13-401

**Published:** 2013-08-30

**Authors:** Afiong O Oku, Eme T Owoaje, Olusimbo K Ige, Angela Oyo-ita

**Affiliations:** 1 Department of Community Medicine, University of Calabar Teaching Hospital, Calabar, Cross river state, Nigeria; 2 Department of Community Medicine, University College Hospital, Ibadan, Nigeria

**Keywords:** Adherence, PLHIV, HAART, Cross river, Nigeria

## Abstract

**Background:**

Adherence to Highly active antiretroviral therapy (HAART) is a major predictor of the success of HIV/AIDS treatment. Good adherence to HAART is necessary to achieve the best virologic response, lower the risk of drug resistance and reduce morbidity and mortality. This study therefore aimed to determine the prevalence and determinants of adherence to HAART amongst PLHIV accessing treatment in a tertiary location in Cross River State, Nigeria.

**Methods:**

A cross-sectional study was conducted among patients on HAART attending the Presidential Emergency plan for AIDS relief (PEPFAR) clinic of the University of Calabar Teaching Hospital between October–December 2011. A total of 411 PLHIV visiting the study site during the study period were interviewed. PLHIV who met the inclusion criteria were consecutively recruited into the study till the desired sample size was attained. Information was obtained from participants using a semi-structured, pretested, interviewer administered questionnaire. Adherence was measured via patients self report and were termed adherent if they took at least 95% of prescribed medication in the previous week prior to the study. Data were summarized using proportions, and *χ*^2^ test was used to explore associations between categorical variables. Predictors of adherence to HAART were determined by binary logistic regression. Level of significance was set at p < 0.05.

**Results:**

The mean age of PLHIV who accessed treatment was 35.7 ± 9.32 years. Females constituted 68.6% of all participants. The self reported adherence rate based on a one week recall prior to the study was 59.9%. The major reasons cited by respondents for skipping doses were operating a busy schedule, simply forgot medications, felt depressed, and travelling out of town. On logistic regression analysis, perceived improved health status [OR 3.11; CI: 1.58-6.11], reduced pill load [OR 1.25; 95% CI: 0.46-2.72] and non-use of herbal remedies [OR 1.83; 95% CI: 1.22-2.72] were the major predictors for adherence to HAART. However, payment for ART services significantly decreased the likelihood of adherence to HAART. [OR 0.46; 95% CI: 0.25-0.87.].

**Conclusions:**

The adherence rate reported in this study was quite low. Appropriate adherence enhancing intervention strategies targeted at reducing pill load and ensuring an uninterrupted access to free services regimen is strongly recommended.

## Background

The Human immunodeficiency virus (HIV) pandemic continues to spread in the population making HIV infection one of the most important public health crises in the world [1]. Globally, about 33.3 million persons were estimated to be infected with HIV/AIDS in 2010, of these, 22.5 million (68%) are in sub Saharan Africa and about 3 million alone in Nigeria. This makes it the country with the second highest burden of HIV and AIDS in the world after South Africa [1]. The current prevalence rate of HIV in Nigeria as at 2010 based on the sentinel surveillance is 4.1%. Cross River State were the study was conducted currently has the 9th highest prevalence (7.1%) in the country as at 2010 and is found in the South-south geopolitical zone of Nigeria [2].

In the absence of a cure, antiretroviral therapy (ART) has remained the only available option that offers the possibility of dramatically reducing HIV/AIDS-related morbidity and mortality, while improving the status of PLHIV. It has proved effective in reducing viral load, improving immune function, [3] and improving the quality of life of PLHIV [3,4]. However, successful long-term treatment of HIV requires strict adherence to the Highly Active Antiretroviral Therapy (HAART) regimen [3,5,6]. This is especially important in countries such as Nigeria where PLHIV make up 10% of the global burden of HIV/AIDS [1] and about 1.5 million require treatment [7]. Adherence level of at least 95% and above has been considered appropriate to achieve therapeutic success, [8-11] as this maintains optimal viral suppression as demonstrated by Paterson and colleagues [11-13]. Failure to observe sustained desired adherence threshold has been associated with dire consequences such as treatment failure, disease progression and emergence of drug resistant HIV/IADS strains [14,15].

With the realization of the central role played by adherence in the success of HIV/AIDS treatment, several studies conducted in various parts of the world including reviews have reported non-adherence rates ranging from 50% to 80% in different contexts [9,10,16]. However, in reality adherence rates are often lower than 95% [11] and rates of adherence from previous studies conducted in Nigeria have ranged from as low as 44% being adherent [17] to more than 95% from different parts of Nigeria [18]. Most of the previous reports were carried out with fewer PLHIV being on treatment restricted access to ARV drugs unavailability and payment for ARV medications were heavily subsidized.

With wide spread awareness of HIV/AIDS, expansion of treatment and prevention programs that have increased ART access to previously un-served and underserved populations in Nigeria coupled with provision of free ART services, [19] there is a need for implementation of continuous monitoring and evaluation mechanisms for adherence, This is of great importance especially because the key to the success of ART programmes and prevention of treatment failures is hinged on consistently high adherence levels. Scaling up of ARVs alone is definitely not the answer when adherence inconsistencies are not tackled. Therefore, the first step to solving this problem is to assess the determinants of adherence to HAART which may differ across geopolitical zones of the country with their unique characteristics of culture, religion, educational status and health seeking behaviours. The objectives of the present study were to determine the prevalence of adherence to HAART and identify factors associated with adherence.

## Methods

The study was conducted at the President’s Emergency plan for AIDS Relief (PEPFAR) clinic now the Specialist treatment clinic of the University of Calabar Teaching Hospital. The Hospital was selected as a centre for the implementation of the President’s Emergency plan for AIDS Relief (PEPFAR) by the United States Agency for International Development (USAID) in June 2005. It has since been responsible for the provision of care and support services for PLHIV as well as one of the major centres where PLHIV both in Cross River and other neighbouring states receive anti retroviral therapy. The patient population is about 4,000, of which more than 2,000 are on treatment. The clinic is run under three units. The paediatric adult and the Prevention of Maternal to Child transmission (PMTCT) clinics.

### Study population

The study population comprised of 411 HIV positive clients who were enrolled and had commenced HAART in UCTH from October 2011- December 2011. They were made of 129 males and 282 females. Participants were consecutively recruited over the study period till the desired sample size was attained. All PLHIV on HAART were eligible to participate except those that satisfied the exclusion criteria. These criteria included PLHIV below 18 years of age, who were attending the clinic but had not commenced HAART, terminally ill patients and pregnant women. The inclusion criteria adopted for the study included consenting out patients diagnosed and confirmed to be HIV positive, at least 18 years of age and had been on HAART for 3 months.

### Study design

A cross-sectional analytical study aimed at documenting the level of adherence among PLHIV on HAART was conducted between October 2011- December 2011.

### Data collection instrument

This consisted of an interviewer administered semi-structured questionnaire which was divided into sections to collect relevant information on socio-demographic data, medical profile including treatment experiences at health facility of respondents. Adherence to HAART in the previous seven days of the interview was measured by self-report. The questions were adopted from The Brief Medication Questionnaire self-report tool for screening adherence and barriers to adherence [20]. The degree of adherence from patient self-reporting was estimated using the following formula: [21]

$$\begin{array}{c} \%\;\mathrm{Adherence}\;\mathrm{over}\;\mathrm{last}\;7\;\mathrm{days}\\ \;=\frac{\#\mathrm{doses}\;\mathrm{should}\;\mathrm{have}\;\mathrm{taken}‒\#\mathrm{missed}\;\mathrm{doses}\times 100\%}{\#\mathrm{Doses}\;\mathrm{should}\;\mathrm{have}\;\mathrm{taken}} \end{array}$$

Then, the percentage of adherence to the antiretroviral was estimated by the average of adherence to the drugs. For the purpose of this study a score of 95% and above represented good adherence and less than 95% was rated as having poor/suboptimal adherence.

### Data analysis

Data were analyzed using SPSS for windows version 19.0. Descriptive and inferential statistical tests were employed. These included bivariate (chi-square) and multivariate (logistic regression) analysis to determine correlates or predictors of adherence. Descriptive statistics (frequencies, proportions, means and standard deviation to summarize variables while Inferential statistics (chi square Test) was used to test the significance of association between categorical variables and level of significance was set at 5%. Logistic regression analysis was used to identify the predictors of adherence to HAART in the study population. Variables entered into the logistic model were those which had earlier been significantly associated on bivariate analysis at 10% significance derived. Predictors were determined at 5% significance.

### Ethical clearance and consent

The ethical Committee of the University of Calabar Teaching Hospital reviewed and approved the study procedures and data collection instruments.

## Results

Four hundred and eleven eligible respondents receiving HAART at the PEPFAR clinic UCTH were studied. The mean age of respondents was 35.7 ± 9.3 years. The largest proportion of the PLHIV 171 (41.6%) were in the age group 25-34 years, followed by 136 (33.1%) in the 35 to 44 age group. The majority of those interviewed were female; 68.6% and currently married 51.8%. More than a third 39.7% had attained at least secondary education and 38.4% were of the skilled non manual occupational group. Also majority 85.9% resided within the state and 67.4% earned less than the minimum wage in Nigeria (<*N*19,000) monthly which is equivalent to $118.75. Almost all (99.8%) respondents interviewed were Christians (Table 1). The Medical profile of respondents interviewed (Table 2) revealed that more, 183 (44.5%) had been on HAART for more than 24 months. The median duration on HAART was 24 months (range 3–192 months). More than half of the study population 226 (55%) had encountered side effects since the commencement of HAART and majority 273 (66.4%) were not using any form of herbal treatment alongside their antiretroviral medications. Furthermore, a little over two-thirds of the respondents 282 (68.6%) were on at least 2 pills per day and a vast majority 386 (93.9%) perceived their health status as improved since their commencement on HAART. A few respondents 49 (11.9%) of the study population reported paying for services at the treatment site.

**Table 1:** **The Socio**-**demographic characteristics of study participants** (**n** = **411**)

**Characteristics**	**Frequency**	**Percentage**
**Age group (years)**		
<25	31	7.5
25-34	171	41.6
35-44	136	33.1
45-54	53	12.9
>55	20	4.9
**Sex**		
Male	129	31.4
Female	282	68.6
**Marital status**		
Single	126	30.7
Married	213	51.8
Divorced	36	8.8
Widowed	36	8.8
**Educational status**		
None	19	4.6
Primary	66	16.1
Secondary	163	39.7
Post secondary	163	39.7
**Occupation**		
Professional/managerial/technical	126	30.7
Skilled manual	41	10.0
Skilled non-manual	158	38.4
Unskilled/unemployed/retired	86	20.9
**Religion**		
Christianity	410	99.8
Islam	1	0.2
**Place of residence**		
Within Cross river	353	85.9
Outside Cross river	58	14.1
**Average monthly income**		
<*N*19,000	277	67.4
≥*N*19,000	134	32.6
**Median(range)**	7000(0–5,000)	

**Table 2:** **Medication**/**treatment variables including treatment experiences** (**n** = **411**)

**Characteristics**	**Frequency**	**Percentage**
**Duration of treatment(months)**		
<12	120	29.2
12-24	108	26.3
>24	183	44.5
**Median duration of treatment(range)**	**24(3**–**192)**	
**Encountered side effects**		
Yes	226	55.0
No	185	45.0
**Herbal use**		
Yes	138	33.6
No	273	66.4
**Number of pills/day**		
≤2	282	68.6
3-4	112	27.3
>4	17	4.1
**Presence of opportunistic infection**		
Yes	32	7.8
No	379	92.2
**Paid for ART services**		
Yes	49	11.9
No	362	88.1
**Perceived health status as improved since commencement of HAART**		
Improved	386	93.9
No improvement	25	6.1
**Perceived health rating**		
Excellent	39	9.5
Very good	253	61.6
Good	92	22.4
Fair/poor	27	6.6
**Transportation cost to Health facility**		
<*N*1000	275	66.9
≥*N*1000	136	33.1

**Figure 1:**
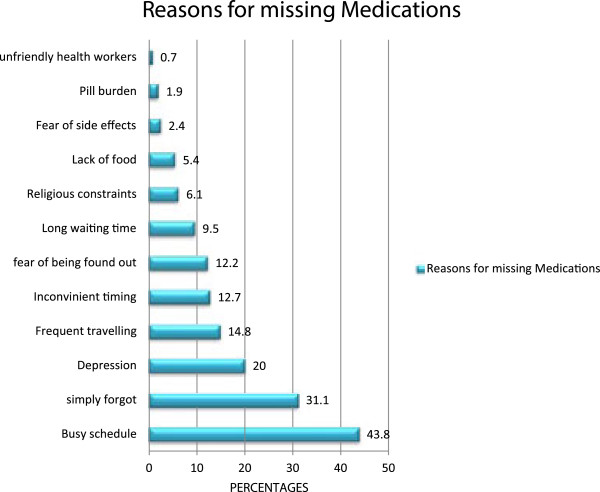
Reasons for missing/skipping medications.

Prevalence of adherence to HAART in the study population showed that more than half (59.9%) of the respondents’ attained 95% adherence to prescribed HAART regimen. The main reasons cited for missing or skipping doses among respondents who missed their medications included being busy (43.8%), simply forgetting (31.1%), depression (20%), frequent travelling (14.8%) and inconvenient timing for medications schedule (12.7%). This is represented in Figure 1.

Factors associated with adherence on bivariate analysis (Tables 3 and 4) were; Being resident outside Cross river state, 42 (72.4%) was significantly more likely to adhere to HAART compared with 204 (57.8%) being resident within the state (p < 0.05). Similarly other significant correlates of adherence included obtaining free ART services, perceived health status as excellent/good and paid more than *N*1000 on transportation to the health care facility (p < 0.05) (Table 4). The predictors of adherence to HAART amongst PLHIV accessing treatment in PEPFAR clinic UCTH were non use of herbal remedies, obtaining free ART services, perceived improvement in health status, and reduced pill load. Participants not using herbal remedies were more likely to adhere to their prescribed doses compared with those using herbal remedies. Similarly obtaining free ART services, taking not more than two pills per day, and a perceived improvement in health following the commencement of HAART than those who paid for ART services, took more than 2 pills per day, and who reported their perceived health status as not improved. This is presented in Table 5.

**Table 3:** **Distribution of socio**-**demographic characteristics**, **of the study participants by their ART adherence pattern**

**Characteristics**	**Good adherence**	**Poor adherence**	**Significance**
	**N = 246**	**(N = 165)**	
	**Frequency (%)**	**Frequency (%)**	
**Age (years)**			
≤35	137(58.3)	98(41.7)	p = 0.46
>35	109(61.9)	67(38.1)	*x*^**2**^ = 0.55
**Sex**			
Male	78(60.5)	51(39.5)	p = 0.864
Female	168(59.6)	114(40.4)	*x*^**2**^ = 0.02
**Marital status**			
Married	133(62.4)	80(37.6)	p = 0.27
Not married	113(57.1)	85(42.9)	*x*^**2**^ = 1.23
**Level of Education**			
None	11(57.9)	8(42.1)	
Primary	36(54.5)	30(45.5)	p= 0.47
Secondary	94(57.7)	69(42.3)	*x*^**2**^ = 2.54
Tertiary	105(64.4)	58(35.6)	
**Residence**			
Within Cross river	204(57.8)	149(42.2)	**p = 0.035**
Outside Cross river Herba	42(72.4)	16(27.6)	*x*^**2**^ = 4.43
**Occupation**			
Professional managerial/Technical	80(63.5)	46(36.5)	
Skilled manual	23(56.1)	18(43.9)	p= 0.74
Skilled non manual	94(59.5)	64(40.5)	*x*^**2**^ = 1.24
Unskilled/unemployed/retired	49(57.0)	37(43.0)	
**Average monthly income**			
≤ N19,000	163(58.8)	114(41.2)	p=0.55
>N19,000	83(61.9)	51(38.1)	*x*^**2**^ = 0.36

**Table 4:** **Distribution of Medical profile**/**treatment experiences of the study participants by their ART adherence pattern**

**Characteristics**	**Good adherence N = 246 Frequency (%)**	**Poor adherence (N = 165) Frequency (%)**	**Significance**
**Use of Herbal remedies**			
Yes	74(53.6)	64(46.4)	p = 0.07
No	172(63.0)	101(37.0)	*x*^**2**^ = 3.36
**Number of pills/day**			
≤**2**	161(57.1)	121(42.9)	p = 0.091
>2	85(65.9)	44(34.1)	*x*^**2**^ = 2.85
**Opportunistic infections**			
Yes	18(56.2)	14(43.8)	p = 0.665
No	228(60.2)	151(39.8)	*x*^2^ = 1.88
**Paid for ART services**			
Yes	21(42.9)	28(57.1)	**p** = **0**.**01**
No	225(62.5)	137(37.8)	*x*^**2**^ = 6.69
**Rating of Perceived Health**			
Excellent/good	238(62.0)	152(39.4)	**p** = **0**.**01**
Fair/poor	8(29.6)	19(70.4)	*x*^**2**^ = 10.1
**Perceived health status**			
Improving	234(60.6)	152(39.4)	p = 0.21
Not improving	12(48.0)	13(52.0)	*x*^**2**^ = 1.56
**Encountered side effects**			
Yes	126(55.8)	100(44.2)	p = 0.061
No	120(64.9)	65(35.1)	*x*^**2**^ = 3.52
**Cost of transportation to health centre**			
<*N*1000 ($ 6.25)	154(56.0)	121(44.0)	**p** = **0**.**023**
>*N*1000 ($ 6.25)	92(67.6)	44(32.4)	*x*^**2**^ = 5.14
**Duration on HAART(months)**			
<24	116(60.1)	77(39.9)	p = 0.864
≥24	130(59.6)	88(40.4)	*x*^2^ = 0.029

**Table 5:** Binary logistic regression analysis of predictors for good adherence

**Independent Variable**	**Odds ratio**	**95% confidence interval**	**p-value**
**Use of herbal remedies**			
No	1.83	1.22- 2.72	**0**.**003**
Yes	1		
**Paid for ART services**			
Yes	0.46	0.25- 0.87	**0**.**017**
No	1		
**Pill number**			
≤2 pills/day	1.84	1.25-2.72	**0**.**002**
>2 pills/day	1		
**Description of perceived health status**			
Improving	3.11	1.58-6.11	**0**.**001**
Not improving	1		
**Residence**			
Outside Cross river	0.76	0.40-1.47	0.42
Within Cross river	1		
**Encountered side effects**			
Yes	0.92	0.65-1.29.	0.61
No	1		
**Transportation costs**			
<*N*1000 ($ 6.25)	0.76	0.50-1.16	0.20
≥*N*1000 ($ 6.25)	1		

## Discussion

The present study aimed to contribute towards addressing gap in knowledge regarding the prevalence and factors associated with treatment adherence among a representative sample of PLHIV accessing treatment site in Cross River State Nigeria.

The self reported adherence reported in the present study was 59.9%. This finding was comparable with other studies done in Nigeria and other African setting, [21-23] but slightly higher than earlier reports by Ilyasu and colleagues in Kano (Northern Nigeria), [18] Nwauche and colleagues (Southern Nigeria) and a study done in Kenya, who reported adherence levels of 54.5%. 49.2%and 43.2% respectively. However adherence level reported in this study was lower than seen in other studies [22,24-26]. Our current findings showed that about two fifths of patients interviewed were non adherent both in terms of dose adherence and timing. Emphasis should therefore be placed on adherence to HAART especially during counselling sessions as this is required for optimal clinical response and complete viral suppression.

The major reasons cited by participants for missing doses included; operating a busy schedule and simply forgetting medications. These two reasons though related to one another have been cited by several studies as the main risk factors for suboptimal adherence [21,23,27-29]. Another important reason given by respondents for skipping or missing medications which is note worthy was feeling depressed. Approximately a fifth of the study population indicated feeling depressed as a reason for poor adherence to medications. This was similar to findings conducted in USA in 2010 where patients with symptoms of depression had higher rates of suboptimal adherence [30]. Issues bothering around stigma and discrimination, personality traits, fear of being discovered, lack of social support, and poorer health outcomes may contribute strongly to emotional non-adjustment to HIV/AIDS, depression and loss of interest in ones treatment. This would ultimately result in poor adherence to medications amongst PLHIV.

Most socio-demographic characteristics such as age, sex, marital status, educational attainment did not significantly affect adherence levels amongst our study population. This corroborates the findings of some authors [17,28,31,32] but refutes the findings of others were certain socio-demographic variables were associated with being adherent to HAART [24,33].

Medication adherence was found to be significantly associated with non use of Traditional herbal medicines. The effect of the use of herbal remedies in addition to HAART was noted in this study. Non herbal use was demonstrated as a significant predictor of adherence in this study. This finding is in agreement with a study conducted in Ilorin, (south western Nigeria) were the use of herbal medicines was a major risk factor for non adherence [34] and a study done in South Africa by Peltzer et al. which demonstrated non use of herbal remedies as a major facilitator of adherence [26]. The reason respondents may consider using herbal remedies could be attributable to the fact that some patients may gradually be losing faith in their anti-retrovirals which most are aware does not provide a cure and are opting for herbal remedies. Secondly, the proliferation of alternative healers and a claim on the media by traditional medicine practitioners of an instant cure for HIV infection may cause some to abandon their treatment.

Pill burden also significantly affected adherence in the present study. Respondents on more than two pills per day were less likely to adhere to their treatment compared to those on at least two pills per day. This finding was also identified as a significant predictor of adherence in a study done by Falang and colleagues in Jos [25] and Sow et al. in Senegal [35] where pill burden was reported to have a strong impact on adherence. Similarly, in India Cauldbeck et al. demonstrated that patients who attained 100% adherence took fewer pills [36]. This could be attributable to the fact that these patients probably because of their busy schedules may have been unable to incorporate their drugs schedule into their daily schedule or may be developing pill fatigue. Reducing pill load as well as dosing schedules to once or twice daily has been found to be associated with better adherence [25].

Self reported improved health status was significantly associated with adherence amongst our study population. The odds of adherence among those who rated their health status as improved was three times compared to those who perceived their health as not improved. This study was in agreement with another study [37] where staying healthy was a key motivator of adherence to treatment. In contrast to our findings, Olowookere and colleagues in south western Nigeria reported that feeling good/healthy, were risk factors for non-adherence. They further reported that most patients tend to abandon treatment once there was an improvement in their health [21,23].

Obtaining free treatment at ART clinics was a significant predictor of adherence to treatment. Respondents who didn’t pay for obtaining ART services were two times better adherers compared to those who paid for services. This was in keeping with studies done in the south western part of Nigeria, [33] and another study done in Botswana when cost were removed as a barrier, adherence was predicted to increase from 54% to 74% [11]. Financial constraints among respondents were a major deterrent to adherence as observed in this study. Although drugs were given free of charge in most health facilities offering ART services, patients are still required to pay some user fees at health facility e.g. opening of folders, certain laboratory investigations and treatment of opportunistic infections. In addition there are some indirect costs these patients bear e.g. cost of transportation to the health care facility. Non-payment of services at every HAART clinics should be enforced and providing free treatment for opportunistic infections will also go a long way in improving adherence.

Certain limitations of this study should be recognized. The cross-sectional nature of the survey did not allow for inferences to be drawn as to causal relationship among variables. The use of self report medication adherence to assess medication adherence was a limitation in this study since participants had to recall their medication adherence in the previous week. Concerted efforts were made to reduce recall bias by limiting recall of medication to 7 days prior to the study which was in itself a limitation because of the possibility that subjects would either over- or underestimated their adherence to HAART. This was further worsened by the inability to corroborate patient self- report of adherence with viral loads and CD4 responses because of financial and logistic constraints of frequent laboratory monitoring. This was because restricted access to patient’s folders.

## Conclusion

This study showed that medication adherence rate was low among PLHIV accessing treatment in UCTH Calabar, Nigeria. It is also obvious that there are barriers to adherence therefore efforts should be targeted and assessed for each patient so that appropriate adherence enhancing interventions can be undertaken. The authors therefore recommend that non-payment of services at every ART clinics and provision of free treatment should also include opportunistic infections and required investigations. This will go a long way in improving adherence. Also the focus during counselling sessions should discourage the use of traditional herbal remedies alongside HAART which has been seen to affect adherence in the present study. In addition, curtailing the activities of Alternative medical practitioners in the country claiming to have found a cure for HIV/AIDS would be of immense help. Pharmaceutical companies involved in manufacture of ARVs should ensure that all recommended ARV regimens should consist of not more than two pills per day.

## Abbreviations

AIDS: Acquired immune deficiency syndrome; ART: Antiretroviral therapy; HAART: Highly active antiretroviral drugs; HIV: Human Immunodeficiency virus; PEPFAR: President’s Emergency plan for AIDS Relief; PMTCT: Prevention of Maternal to Child transmission; PLHIV: People living with HIV/AIDS; UCTH: University of Calabar Teaching Hospital; USAID: United States Agency for International Development.

## Competing interests

The authors declare that they have no competing interests.

## Authors' contributions

OA conceived, designed and coordinated the study, she carried out statistical analysis and drafted the manuscript; OE contributed by means of her competence and experience in reviewing the manuscript critically for its intellectual content and also participated in the conception and design of the study. IO and OA participated in the design of the study, questionnaire and critically reviewed the manuscript. All authors read and approved the final manuscript.

## Pre-publication history

The pre-publication history for this paper can be accessed here:

http://www.biomedcentral.com/1471-2334/13/401/prepub
